# Feasibility of a Hospital Information System for a Military Public Organization in the Light of the Multi-Criteria Analysis

**DOI:** 10.3390/healthcare10112147

**Published:** 2022-10-28

**Authors:** Ruan Carlos Alves Pereira, Miguel Ângelo Lellis Moreira, Igor Pinheiro de Araújo Costa, Fabrício Maione Tenório, Naia Augusto Barud, Luiz Paulo Fávero, Anas Ali Al-Qudah, Carlos Francisco Simões Gomes, Marcos dos Santos

**Affiliations:** 1 Production Engineering Department, Federal Fluminense University, Rio de Janeiro 24210-240, Brazil; 2 Operational Research Department, Naval Systems Analysis Centre, Rio de Janeiro 20091-000, Brazil; 3 School of Economics, Business and Accounting, University of São Paulo, Sao Paulo 05508-010, Brazil; 4 Faculty of Business, Liwa College of Technology, Abu Dhabi 51133, United Arab Emirates; 5 Systems and Computing Department, Military Institute of Engineering, Rio de Janeiro 22290-270, Brazil

**Keywords:** hospital information systems, public healthcare, multi-criteria decision analysis, THOR, PROMETHEE, decision support systems

## Abstract

The healthcare environment presents a large volume of personal and sensitive patient data that needs to be available and secure. Information and communication technology brings a new reality to healthcare, promoting improvements, agility and integration. Regarding high-level and complex decision-making scenarios, the Brazilian Navy (BN), concerning its healthcare field, is seeking to provide better management of its respective processes in its hospital facilities, allowing accurate control of preventive and curative medicine to members who work or have served there in past years. The study addresses the understanding, structure and clarifying variables related to the feasibility of technological updating and installing of a Hospital Information System (HIS) for BN. In this scenario, through interviews and analysis of military organization business processes, criteria and alternatives were established based on multi-criteria methodology as a decision aid. As methodological support for research and data processing, THOR 2 and PROMETHEE-SAPEVO-M1 methods were approached, both based on the scenarios of outranking alternatives based on the preferences established by the stakeholders in the problem. As a result of the methodological implementation, we compare the two implemented methods in this context, exposing the Commercial Software Purchase and Adoption of Free Software, integrated into Customization by the Marine Studies Foundation, as favorable actions to be adopted concerning HIS feasibility. This finding generates a comprehensive discussion regarding the BN perspective and changes in internal development in the military environment, prospecting alignment to the culture of private organizations in Information Technology for healthcare management. In the end, we present some conclusions concerning the study, exploring the main points of the decision-making analysis and for future research.

## 1. Introduction

The technological evolution of recent years has established new trends in the corporate world, where innovations made possible by technology have allowed the emergence of various business models, bringing about the urgency of digital transformation in the organization [1]. The industry and services of the information technology (IT) sectors have been promoting a new reality regarding improvements, agility, data processing and systems integration, often presenting data and information as the most critical assets of a given organization [2].

Specifically, concerning the health area, the implementation of information management systems has been presented as of tremendous value [3], providing data processing as a form of support in managing public and private health systems [4]. For Gandarillas and Goswami [5], this process facilitates the development of new and more effective monitoring, prevention and treatment approaches, which were impossible in earlier years. Health technology assists the patient and gives health professionals a practical and reliable resource with which to perform their work [6].

As presented in [7], the integration of IT into health systems has been consolidated as a new research environment and science that studies how the use and proper treatment of information can improve the quality of health services provided to patients [8], increase productivity and facilitate access to knowledge [9]. It is also emphasized that information management in the health area is not restricted to administrative processes but applies to planning health services and clinical and epidemiological research [10,11].

Information management in public environments is complex and should be strategic [12], supporting resource management and decision-making [13,14]. As motivation for this study, the Brazilian Navy has been dedicated to improving its respective information systems for health management, seeking the centralization of systems and parameterization of databases on military health history. Thus, the following question: How to enable data processing and information management through an integrated technological system with high reliability for monitoring military personnel health in the Brazilian Navy?

For the analysis of problems of high complexity involving multiple variables, Operational Research (OR) in the form of science enables, through its approaches and methodologies, the analysis of problematic situations based on technical and scientific means [15], making possible the structuring, understanding and clarification of a favorable solution for a given problem [16]. It is emphasized that the models present in the OR field are not restricted to applying an equation [17], but rather to the implementation of axiomatic models, integrating logic and a mathematical basis in the modeling of the treatment of the problem [18].

Considering the problematic situation of enabling an IT health system within the Brazilian Navy, it is necessary to consider variables influential to the analysis [19]. However, with the involvement of multiple contexts and circumstances, the increase in complexity in a given analysis becomes noticeable [20], with different points of view regarding the preference for or influence of a decision variable [21]. However, it is necessary to consider this in terms of a substantial evaluation and greater assertiveness in the final decision [22,23].

Related to the analysis of complex problems, Multi-Criteria Decision Analysis (MCDA) is a field of OR that provides structuring of the problem variables, enabling the analysis of complex problems of evaluation of different types, considering risk and uncertainty in a transparent format [24]. According to Devarakonda et al. [25], the methods present in the MCDA establish preferences between the alternatives under a set of multiple criteria, presenting these different levels of importance, priorities and conflict [26].

When evaluating real and complex problems, aspects of uncertainty and subjectivity are intrinsic [27], being necessary for the modeling used, enabling the treatment of this information in favor of a substantial evaluation closer to reality [28]. Enabling the treatment of subjectivity in a multi-criteria analysis is essential, enabling the transcription of decision-making preferences related to manipulations and attributions performed in the evaluation of the problem, often operating hybrid models, integrating and processing data of both qualitative and quantitative nature [29].

Concerning the BN environment, the problematic situation addresses the complexity in providing an analysis that clarifies assertive action to be adopted in terms of the strategic feasibility of a Hospital Information System (HIS) for its healthcare system. The contextualization integrates questions, such as: Which is the most profitable strategy for development? Which criteria set can be adopted with significant influence on the analysis? Purchase a commercial technology, or provide an intern product? Which level of security is necessary to be integrated? Questions like these reveal the motivation of this study, where we seek to present the structuring of variables and their correlation to stakeholder preferences.

In this scenario, the study is based on a decision-making analysis, implementing HIS for the Brazilian Navy. To support the decision-making process, the multi-criteria approach is used through the THOR 2 method [30,31] and PROMETHEE-SAPEVO-M1 [32], both methodologies being state of the art, treating variables of different natures and enabling an analysis of sensitivity to the problem under evaluation. The flexibility offered by the methods justifies its implementation, where a problem with qualitative and subjective perspectives can be integrated into quantitative data for a more accurate analysis of health systems.

In favor of an assertive and simple implementation, it uses its web computing platforms, enabling the exploration of numerical and graphic resources in the evaluation. Due to the necessity of integrating numerical and subjective data, the latter as tacit knowledge, the THOR 2 and PROMETHEE-SAPEVO-M1 were selected from a set of MCDA approaches, making possible the integration of quantitative and qualitative information in an axiomatic procedure, both of them transcribing the results in a cardinal ranking of variables.

The article is divided into five sessions. After contextualizing the study in the introduction, Section 2 explores the theoretical foundations of the multi-criteria approach, presents a bibliometric analysis and specifies the axiomatic structure of the methods applied. Section 3 presents the case study, exploring the problem under investigation, numerical application of the methods used as support basis, analysis of results and comparisons. Finally, Section 4 discusses the approach addressed and Section 5 presents the final considerations of the study and proposals for future research.

## 2. Theoretical Background

Decision-making is one of the fundamental tasks of management, understood as a way of achieving goals or organizational objectives, correctly expressing objectives, establishing different forms of solution [33], analyzing their feasibility and consequences and seeking the resolution of problems by implementing the most favorable solution according to the decision-making agent [34,35].

Making decisions about hospital resource management is not trivial and incorrect decision-making can severely affect the quality of healthcare services provided to the community [36]. 

Since hospital service evaluation involves many qualitative and quantitative factors, it is a complex MCDA issue [37]. Innovative approaches to assessing and managing medical technologies use a combination of health technology assessment (HTA) and operations research methods, specifically MCDA [38].

The HIS is a computer system that provides a paperless environment that covers all aspects of the hospital operation, including clinical, administrative and financial [39].

As explored in [40], the decision-making process is the main essence of management and can be understood as a set of steps that make up the whole process to be carried out [41]. The MCDA methods, concerning the context of decision-making processes, seek to enable the decision-making process to be as neutral, objective and transparent as possible, enabling the analyst to better understand the structure under evaluation and obtain a reasonable and favorable solution [40].

Recognized as decision support tools, MCDA methods are relatively important within the OR due to their robustness and flexibility in treating and evaluating complex problems. In general, the MCDA models seek to establish preference relationships between the elements of a set of alternatives under the light of a set of criteria present in a decision-making process. It is significant that one of the great merits of data models is to provide, through methodologies and algorithms, the treatment of subjectivity intrinsic to decision-making analysis, supporting the decision-making in a reasonable explanation of preferences [17,42].

The academic literature presents some studies on multi-criteria analysis in hospital management. For example, Vahidnia et al. [43] considered the specific problem of creating a well-distributed network of hospitals that delivers its services to the target population with minimal time, pollution and cost. The authors developed an MCDA process combining Geographical Information System (GIS) analysis with the Fuzzy Analytical Hierarchy Process (FAHP) to determine the optimum site for a new hospital in the Tehran urban area.

Bilsel et al. [44] applied the AHP and PROMETHEE (Preference Ranking Organization Method for Enrichment Evaluation) methods to measure the performance of the websites of Turkish hospitals. Liao et al. [45] proposed a linear programming method to solve MCDA problems. The framework was applied in the evaluation of hospitals.

Liu et al. [46] present an evaluation of hospital waste disposal alternatives that can be considered a complicated MCDA problem that requires the consideration of several alternative solutions and conflicting tangible and intangible criteria; in the research in question, a new MCDA technique based on fuzzy set theory is presented and the VIKOR method is used to evaluate disposal methods.

Nilashi et al. [39] developed a model to determine the most important factors among the four categories for HIS adoption in the context of Malaysian public hospitals. The elements were identified and compared by 20 hospital experts and decision-makers. The authors applied the fuzzy ANP method to compute the weights of incorporated factors in the HIS adoption. The results revealed that hospitals with compatibility, complexity, mimetic pressure and vendor support were more likely to adopt HIS. Hence, the decision to adopt HIS was mainly determined by technological and environmental context. 

Karagiannidis et al. [47] discussed treatment practices for infectious hospital wastes in Central Macedonia through the AHP method. The analysis demonstrated that a centralized autoclave or hydro-clave plant near Thessaloniki was the best performing option, depending on the selection and weighing of criteria of the multi-criteria process. 

Amaral and Costa [36] applied the PROMETHEE II method to support decision-making and resource management in an Emergency Department (ED). The PROMETHEE was chosen for this study because its outranking approach is considered appropriate for the decision-making context of hospital services. The ranking showed the best alternatives to be implemented to improve the throughput of patients in the “Blue Room”. 

Akdag et al. [48] applied the AHP and TOPSIS methods to evaluate the service quality of some Turkish hospitals. The study found the importance and weights of performance criteria with AHP, while TOPSIS was applied to find and rank efficient performance values. 

Senvar et al. [49] handled the problem of establishing a well-organized and distributed hospital network that delivered its services to the target population. The authors proposed a new MCDA method, integrating hesitant fuzzy sets (HFSs) into the TOPSIS method. The proposed methodology was implemented to select the optimum site for a new hospital in Istanbul.

Ivlev et al. [38] determined a ranked list of Magnetic Resonance Imaging (MRI) systems for contributory health organizations administered by regional authorities (regional hospitals) in the Czech Republic. The authors used the AHP and Delphi methods to identify experts’ preferences and for consensus building. 

Fei et al. [37] extended the traditional best-worst method (BWM) based on belief function theory (BFT). Based on the constructed model, the authors presented a case study to evaluate hospital service quality. 

Cardoso et al. [50] performed a suspect screening analysis of 2030 pharmaceuticals and metabolites in hospital effluent samples, applying different sample preparation techniques. Additionally, the authors identified contaminants with the ELECTRE multi-criteria decision analysis technique, making it possible to prioritize analytes according to their environmental risk and enable their inclusion in environmental monitoring programs.

Taking into account these previous studies, the study in question seeks to apply two state of the art methodologies, enabling the comparison of the results generated by these different axiomatic models, and promoting treatment and transparency regarding the perception of decision-makers in the decision-making process. In this context, the research to be explored in the following sections seeks to expose the alignment of the problematic situation under discussion, which is favorable to analysis based on the two MCDA models to be explored.

### 2.1. Bibliometric Analysis

In the search to present the significance of this study, bibliometric research was carried out in the Scopus database, limited to the period from 2000 to 2022, seeking clarification of the importance of the theme in question. As a search, the term “Hospital Information System” was used in a Scopus database search. To support the analysis, Bibliometrix software was used [51], processed by R computational language [52].

**Figure 1:**
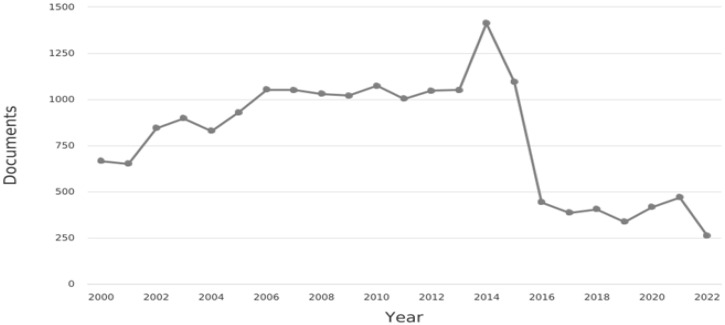
The number of publications between 2000 and 2022.

The given research presented a total of 18,375 documents distributed among scientific articles, books, etc. As shown in Figure 1, the theme exposes significant relevance, providing a favorable volume of annual publications and exposing a high degree of interest and relevance in the academic community.

Another vital factor is aligned with the application area, presenting a significant presence in scientific research for medicine, health management, engineering and computational sciences. When considering the countries with the highest numbers of surveys dedicated to the given theme, a high point is observed in the United States, having approximately 30% of the studies indexed in the Scopus database. Following, however, possessing approximately 5% of the total, are China, Germany and United Kingdom. The other countries have between 2% and 3% of the total publications.

In addition, a filter was performed in the searches, specifying the applications strictly directed to using multi-criteria support for decision-making. This scenario identified 72 papers, this time exposing an increase in the number of publications per year. It is emphasized that the low number of publications, from MCDA to HIS, highlights the need for an increase in the number of studies related to the given theme.

**Figure 2:**
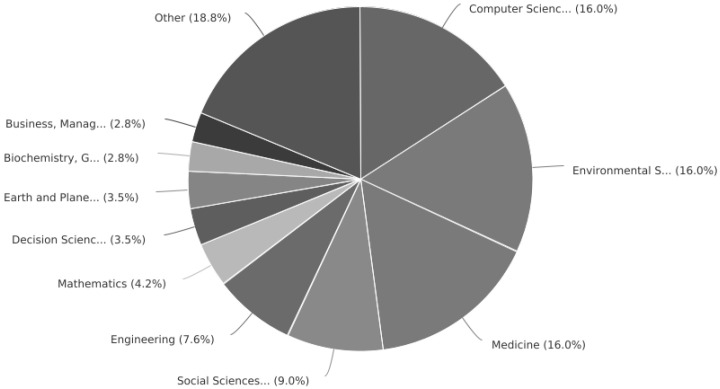
Areas of publication with multi-criteria.

As for application areas, there is a lower concentration of studies in a single area, where medicine, systemic environments and computer science share the same proportion of publications, preceded by engineering, decision sciences and mathematics. Figure 2 exposes the areas of the various publications. 

**Figure 3:**
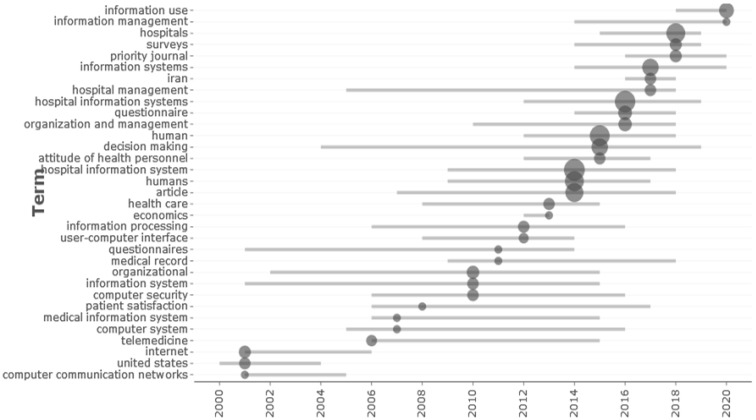
Main journals related to the theme.

Finally, the main research topics related to the theme were observed, thus exposing the periods per year in which a concentration of these specific applications was identified via multi-criteria. These terms present significant areas of study to focus efforts on future research concerning healthcare systems. Figure 3 shows the data.

### 2.2. THOR 2 Method

The THOR 2 method consists of the axiomatic evolution of THOR, based on the integration of preference modeling (which brings it closer to the French School) and multi-attribute and applicable theories (which brings it closer to the American School). These theories allow the attractiveness of an alternative to be quantified by creating a non-transitive aggregation function. As presented, the given modeling provides the following contributions:

1. It presents a hybrid algorithm that simultaneously aggregates concepts of the theory of approximate sets (RST), theory of cloudy sets, utility theory and modeling of preferences;
2. Sorts discrete alternatives to intransitive or non-transitive decision-making processes;
3. It eliminates redundant criteria, considering simultaneously whether the information is dubious (use of RST) and whether there is an increase in the inaccuracy of the decision-making process (use of cloudy set theory);
4. Quantifies inaccuracy and uses it in the MCDA decision-making process;
5. It allows the entry of data from more than one decision-taker simultaneously, allowing them to express their judgment(s) of value(s) on the scale of reasons, intervals, or ordinals;
6. The new formula used by THOR (for weight assignment on the ordinal scale) was obtained after the study of the three formulas existing in the literature;
7. The decision-taker can also execute the decision-making process without assigning weights to the criteria;
8. It eliminates the need for some algorithms based on modeling preferences to determine a value, typically arbitrary, for agreement.

The following additional elements may be required for THOR application: (i) weight for each criterion, representing the relative importance between them; (ii) one preference threshold (*p*) and another for indifference (*q*) for each criterion; (iii) a definition of the domain of disagreement; (iv) characterization of the pertinence of the values of the weights attributed to the criterion; (v) the relevance of the classification of the alternative in the criterion. It should be emphasized that the relationships achieved through THOR have a numerical quantity representing the value of the alternative. This is accomplished through an additive value function. The relationship of dominance and the hierarchy of the values of the alternatives are thus constructed. Three situations allow one alternative to be better than another [30]. Equations (1)–(3) demonstrate the three situations that allowed for one alternative to be considered preferable to the other.

(1) $$S1:\;\overset{n}{\underset{j=1}{\sum }}\left(w_{j}|aP_{j}b\right)>\overset{n}{\underset{j=1}{\sum }}\left(w_{j}|aQ_{j}b+aI_{j}b+aR_{j}b+bQ_{j}a+bP_{j}a\right)\;$$

(2) $$S2:\;\overset{n}{\underset{j=1}{\sum }}\left(w_{j}|aP_{j}b+aQ_{j}b\right)>\overset{n}{\underset{j=1}{\sum }}\left(w_{j}|aI_{j}b+aR_{j}b+bQ_{j}a+bP_{j}a\right)\;$$

(3) $$S2:\;\overset{n}{\underset{j=1}{\sum }}\left(w_{j}|aP_{j}b+aQ_{j}b\right)>\overset{n}{\underset{j=1}{\sum }}\left(w_{j}|aI_{j}b+aR_{j}b+bQ_{j}a+bP_{j}a\right)\;$$

Equation (1), concerning the first scenario, addresses a strict performance of alternatives, considering the aggregation of only the punctuation obtained through strict preference between alternatives in each criterion. Regarding the second scenario, Equation (2) is considered an aggregation of the degrees of strict and weak preference. Finally, addressing a more flexible evaluation, the third scenario in Equation (3) integrates all possible positive degrees obtained; in this way, S3 considers strict preference, weal preference and indifference punctuation.

Equations (4)–(6) consider the preferred relationships *P* (strict preference), *I* (indifference) and *Q* (weak preference), considering the limits of preference and indifference:

(4) aPb ↔ g(a) − g(b) > p

(5) aIb ↔ −q ≤ |g(a) − g(b)| ≤ q

(6) aQb ↔ q ≤ |g(a) − g(b)| ≤ P

**Figure 4:**
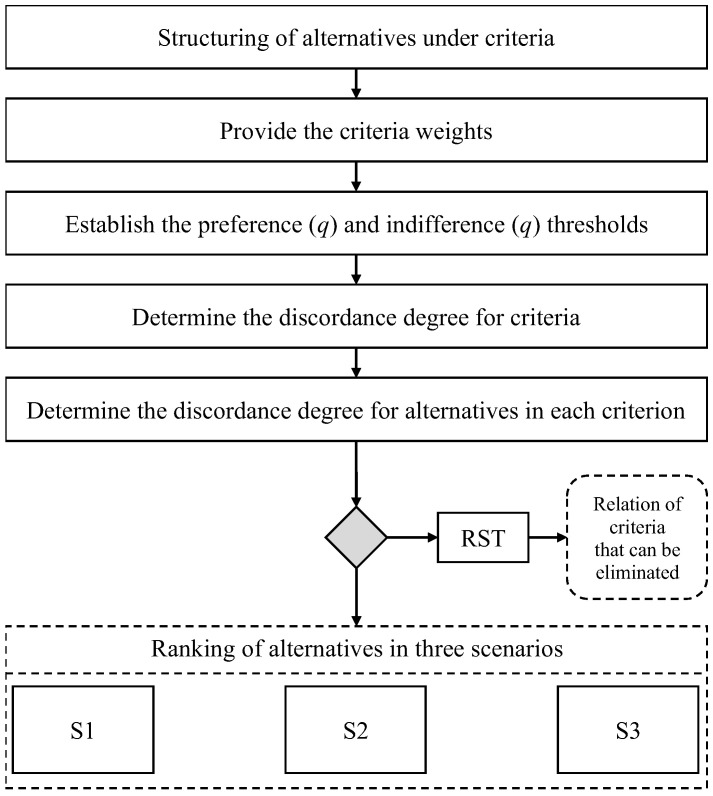
Axiomatic structure of the THOR method.

The THOR 2 method distinguishes the attribution of weights in situations of indifference and weak preference in scenarios S1, S2 and S3. Situations with indifference bring with them half the weight value of the respective criterion. The differences in weak preference carry a ratio between half the value of the criterion weight and the total weight value. A graphical description of the THOR 2 method is shown in Figure 4.

As presented in Figure 4, the methodology starts by defining the variables of a problematic situation, in this context the set of criteria and alternatives under each criterion. Following the process, each criterion is evaluated, building its weight of importance. Each criterion establishes an indifference and preference threshold, in other words, an inferior and superior limit for the normalization process of alternatives. Then the degrees of discordance for criteria and alternatives in each criterion is determined, providing a better performance of variables in the analysis. In the end, three different scenarios for analysis are explored, addressing a strict and flexible evaluation from S1 to S3, respectively. 

Regarding the RST process, the analysis is only carried out by the necessity exposed by the decision-maker, where some criteria can be indicated as redundant, therefore removing a criterion and performing a new analysis of alternatives, trying to identify some rank reversal problems aligned to the dependence between criteria. 

The THOR 2 method also provides that the value of the criterion weight is multiplied by the cloudy-approximation index, deteriorating the comparison according to the degree and safety of the data [53]. Thus, even with lack of data, the method allows the completion of the classification of alternatives and weights in the decision matrix, making it possible to estimate the value and assign a low value of pertinence to the attribution of that data, thus avoiding the elimination of the alternative or criterion due to the absence of data. Unlike the previous version of the model, THOR 2 proposes that, in situations where strict preference, weak preference and indifference occur, the weight value is multiplied by the cloudy-approximation index, fully contemplating the uncertainty of the model. In the first version of the THOR method, the weight value deteriorates only in the weak preference situation [30]. 

THOR 2 has already been successfully applied to select the most suitable hospital care vessel for BN to support the COVID-19 pandemic. The method allowed a robust sensitivity analysis, giving greater transparency and reliability to the decision-making process [54].

To expand the use of the methods by the scientific community, software was developed for the THOR and THOR 2 methods, called THOR Web.

### 2.3. PROMETHEE-SAPEVO-M1

The integration of MCDA methods characterizes the PROMETHEE-SAPEVO-M1 modeling proposed by Moreira et al. [32]: PROMETHEE (Preference Ranking Organization Method for Enrichment Evaluations) [55] and SAPEVO-M (Simple Aggregation of Preferences Expressed by Ordinal Vectors—Multi Decision Makers) [56]. 

The modeling is presented as a non-compensatory methodology, handling ranking problems and evaluating a set of alternatives under multiple criteria, performed in the classical PROMETHEE method [57]. The method establishes a preference structure between the alternatives, considering a preference function defined by the decision-maker for each criterion, where the global index enables the outranking of the alternatives [58].

Integrated into the method, the techniques presented in the SAPEVO-M method [56] approach as ordinal evaluation transcribed by linguistics terms, providing a cardinal index upon an aggregation procedure based on the pairwise comparison, aiming to express the respective preferences of the decision-maker [59].

**Figure 5:**
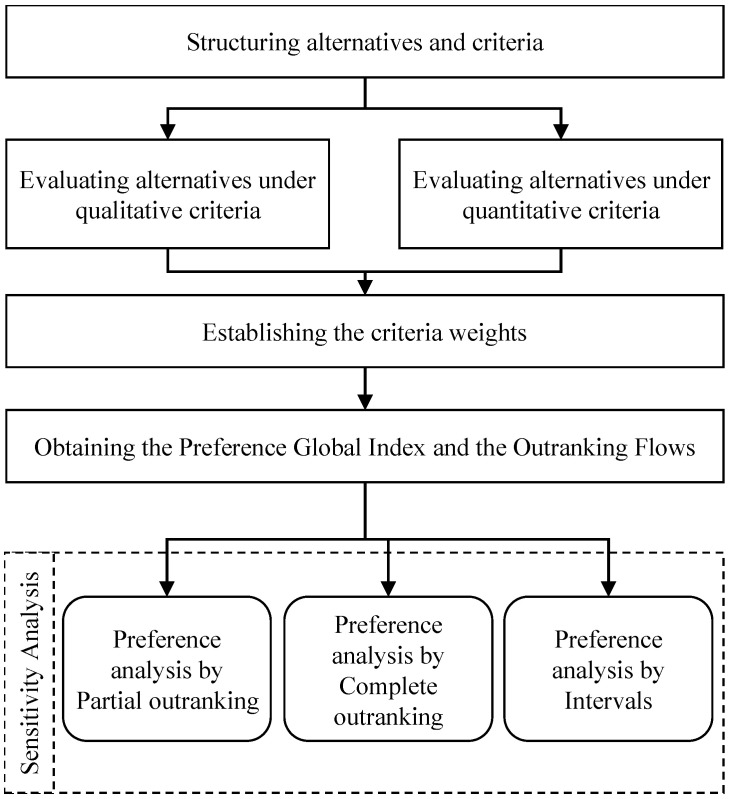
Axiomatic structure of the PROMETHEE-SAPEVO-M1 method.

Thus, the PROMETHEE-SAPEVO-M1 method allows a multi-criteria evaluation through a non-compensatory algorithm for ranking problems, considering quantitative and qualitative variables through cardinal and ordinal inputs [32]. Operating as a hybrid method enables the manipulation of both natures of criteria or, if necessary for the problem, just one type. The main characteristic of the method concerns the possibility of providing a sensibility analysis of a problem by three models of preference analysis, partial, complete and intervals [60]. The axiomatic structure is displayed in Figure 5.

The axiomatic process establishes alternatives and criteria, which can be divided into qualitative and quantitative. Each nature has a different axiomatic process, detailed in the study [32]. Each criteria analysis obtains a performance matrix between the alternatives, exploring the pairwise performance among two variables. Then the process is guided by obtaining criteria weights through pairwise analysis and performing the building preferences degrees for each criterion. In the end, the global preference index is calculated, indicating a performance matrix between each pair of alternatives considering all punctuation in the aggregated criteria.

As presented by Moreira et al. [58], with the implementation of the method, positive (7), negative (8) and net (9) flows are generated indicating the performance of alternatives concerning the decision-maker preferences. The positive flow 𝜙+ (7) represents how an alternative *a* outranks the other alternatives in the set and the negative flow 𝜙− (8) represents how *a* is outranked by the other alternatives. The higher the positive flow and the lower the negative flow, the more preferable the alternative. Moreover, the net outranking flow 𝜙 (9) is presented, representing the difference between the positive and negative flows, providing a complete ranking.

(7) $$\phi^{+}\left(a\right)=\frac{1}{n-1}\underset{x\in A}{\sum }\pi \left(a,x\right)$$

(8) $$\phi^{-}\left(a\right)=\frac{1}{n-1}\underset{x\in A}{\sum }\pi \left(x,a\right)\;$$

(9) $$\phi \left(a\right)=\phi^{+}\left(a\right)-\phi^{-}\left(a\right)$$

Performing the positive outranking, characterized by the global preference of alternative a over all other alternatives from the set, and the negative outranking, represented by the preference of all alternatives over alternative a, it is possible to obtain a partial ranking of variables. As addressed in [32], this analysis reflects the PROMETHEE I evaluation, where the higher is the positive and the lower the negative index, the better the alternative, as presented in (10)–(12):

▪ a is preferable to $b\;\left(aPb\right)$ *if*:
  (10) $$\left\{\begin{array}{c} \phi^{+}\left(a\right)>\phi^{+}\left(b\right)\;and\;\phi^{-}\left(a\right)<\phi^{-}\left(b\right),\;or\\ \phi^{+}\left(a\right)=\phi^{+}\left(b\right)\;and\;\phi^{-}\left(a\right)<\phi^{-}\left(b\right),\;or\\ \phi^{+}\left(a\right)>\phi^{+}\left(b\right)\;and\;\phi^{-}\left(a\right)=\phi^{-}\left(b\right);\; \end{array}\right.\;$$
▪ a is indifferent to b $\left(aIb\right)$ *if*:
  (11) $$\phi^{+}a=\phi^{+}\left(b\right)\;and\;\phi^{-}\left(a\right)=\phi^{-}\left(b\right)\;$$
▪ a is incompatible to b $\left(aRb\right)\;$*if*:
  (12) $$\left\{\begin{array}{c} \phi^{+}\left(a\right)>\phi^{+}\left(b\right)\;and\;\phi^{-}\left(a\right)>\phi^{-}\left(b\right),\;or\\ \phi^{+}\left(a\right)<\phi^{+}\left(b\right)\;and\;\phi^{-}\left(a\right)<\phi^{-}\left(b\right);\; \end{array}\;\right.$$

The complete outranking is generated by the net flows, whereas the higher the index, the better the alternative in this analysis, generating relations of preference and indifference between the variables, as shown in (13) and (14):

▪ a is preferable to b $\left(aPb\right)$ *if*
  (13) $$\phi \left(a\right)>\phi \left(b\right)\;$$
▪ a is indifferent to b $\left(aIb\right)$ *if*
  (14) $$\phi \left(a\right)=\phi \left(b\right)\;$$

The third model of preference analysis associates with each alternative net flow an interval $[x,y]$, based on an expected error value from the sample set of net outranking flows, where each interval is obtained by Equation (15). This model of analysis enables definition of the complete ranking, (16) and (17):

(15) $$\left\{\begin{array}{c} x_{a}=\phi \left(a\right)-\alpha \sigma_{a}\\ y_{a}=\phi \left(a\right)+\alpha \sigma_{a} \end{array}\right.$$

▪ a is preferable to b $\left(aPb\right)$ *if*
  (16) $$x_{a}\leq y_{b}\;and\;x_{b}>y_{b}$$
▪ a is indifferent to b $\left(aIb\right)$ *if*
  (17) $$\phi \left(a\right)=\phi \left(b\right)$$

Analyzing the set of variables by different manipulation models makes it possible to realize a sensitivity analysis of the problem, performing many results and clarifying the preferences in different evaluation scenarios [32]. To assist in the method implementation, a web platform has been developed [61], enabling the understanding and application of the model and presenting a friendly interface through its numerical and graphical analysis.

## 3. Case Study

To provide medical assistance to the military of the BN and their families, given that the military organization has a set of hospitals, clinics and hospital ships in constant operation destined for the care and health treatment of members linked to BN, it is necessary to have a vast care network, since the military is distributed throughout the country.

In this context, the need to enable a HIS that integrates the hospital networks and administrative and health management of the members linked to the care centers was identified. Thus, an exploratory study of the problematic situation was carried out, seeking to provide a better understanding of the fundamental needs to be achieved by the BN regarding the integration of a HIS into its health system.

With the help of a group of BN officers working within the IT field, a series of meetings and interviews were held, both with IT managers and managers of BN hospitals, providing the survey with influential criteria for a given problem along with all possible forms of solutions to be implemented. In the group composed of five officers, there were three officers with more than 20 years of experience in the development of specific systems for BN. The other two officers performed activities in managing BN hospitals for more than 10 years, operating directly in their current HIS.

The questions operated in the interviews aimed to clarify the main variables of influence and dependence on the acquisition or development of a HIS. We emphasize that the interaction model was conducted through individual interviews in the first moment and then a group evaluation to determine the variables of higher value for managers.

Initially, BN had proposed using criteria cost, implementation time and technological dependence. However, after the exploratory analysis, five more elements were defined, totaling eight criteria, clarified as the variables with the most significant influence on implementing a HIS. The set of criteria is described in sequence:

1. Adherence to business processes: Considered the most important criterion, this refers to how HIS will operate to meet the respective needs of users, with all functionalities, fields, permissions and forms, among other elements, adherent to the reality of BN;
2. Term: Time is a scarce resource in IT and, when it comes to health, the theme becomes even more critical and can be characterized as one of the most significant restrictions in the development of a computer system;
3. Customization: The customization criterion is related to the software ability to adapt to new processes and regulations, thus demanding a respective level of flexibility of the model developed concerning organizational needs;
4. Cost: This is a variable directly linked to the expenses and feasibility of the project in question, representing the amount of investment necessary for the implementation of HIS;
5. Need for specialized labor: The development of a technological model needs to be prepared to support applications, to correct errors, provide information security and enable the maintenance of systems, so having a skilled and specialized workforce is another factor of extreme importance within this context, from the moment that the decision whether HIS will be developed externally or internally will involve the choice of using a workforce from within or outside the organization;
6. State of the art: This dimension is associated with the alignment of the organization with the best that the current market can offer so that the system does not become stagnant as technology evolves;
7. Technological dependence: Given that this variable is related to the dependence of technologies external to the organizational environment, presenting the relationship with the supplier of specific technology can limit what can be adapted in the system during and after the implementation.
8. Vulnerability to errors: the given criterion represents the maturity of a computational system, from the moment from which shelf models can represent greater maturity of technology than newly developed software.

Based on the set of criteria, a set of alternatives was defined based on four types of generalist actions:

1. Develop internally: all requirement gathering is done from scratch, with all needs, interfaces, screens, reports, integrations and functionalities, in this case, which makes the development the responsibility of a team from the institution itself;
2. Develop externally: all requirement gathering is done from scratch, with all needs, interfaces, screens, reports, integrations and functionalities; in this case, who carries out the development is an outsourced company contracted to create the software on demand;
3. Shelf software: buy on the market software ready, standardized and non-customizable, with the definition of minimum requirements for adequacy;
4. Customizable software: buy on the market software ready and standardized, but customizable if necessary.

Considering the variables in the problem, for the choice of the most favorable method of implementing HIS for BN, six types of alternatives were considered, divided into alternatives of internal development and external development:

External development:

1. Commercial Software Purchase (CSC)
2. Adoption of Free Software + Customization by BN (SL + BN)
3. Adoption of Free Software + Customization by the Marine Studies Foundation (SL + FEM)

Internal development:

4. Development by BN (DBN)
5. Development by the Foundation for The Studies of the Sea (DFEM)
6. Development by Outsourced Software Factory (DFST)

All data were obtained through quotations from companies in the market with documentation of BN technological development. Calculations related to the costs of technologies were considered: purchase, development, maintenance, implementation, training and labor involved. No infrastructure costs, development tool/bank/virtual machine licenses, or other deployment costs were considered.

It was also considered that, when purchasing commercial software or adopting a free software solution, the decision-taker is aware that these are software produced by third parties and that they have defined pre-established processes according to the scenario of their respective institutions [62]. Any adaptations to meet the BN processes must be raised and developed before effective implementation.

The six alternatives were analyzed based on the management of the eight criteria for projects implementing automated systems in BN clinics, considering the most experienced and qualified experts to perform these evaluations. The decision-makers performed other evaluations and measurements based on their experience, BN expectations regarding the project and business knowledge. 

The decision matrix for the given problem is presented in Table 1, indicating the quantitative values for the set of alternatives in each criterion: Cost (C1), Time (C2), Technological Dependence (C3), Need for SL (C4), Adherence (C5), Customization (C6), state of the art (C7) and Vulnerability (C8). The feasibility expenses in Reais (R$) were used for the cost criterion. For the deadline, the necessary months are used to make the system viable; technological dependence is indicated by the percentage of involvement of the outsourced organization to BN; Labor Need represents the number of people involved in the project; and finally the remaining criteria operate in an evaluation determined on a seven-point scale, where the higher the score indicated, the more favorable the alternative for that given criterion.

The MCDA THOR 2 and PROMETHEE-SAPEVO-M1 methods analyze a given problematic situation. Both models will be implemented separately and a comparison analysis between the implemented models subsequently carried out. We highlight that the implementation of two methodologies does not address identification of which method can be more applicable but makes it possible to provide an overview of logical alignment, where different axiomatic models can present similar or contradictory results by manipulation of the same dataset.

### 3.1. THOR 2 Numerical Implementation

The information passed by the decision-taker and the dataset were considered in implementing the model for the problem. In this context, the weight values of the criteria were defined together with the attributions of the q and p values for each criterion. In this way, Table 2 presents the respective data.

Considering the aggregation of preferences, thresholds and weights of the criteria, the result was obtained for the performance of the alternatives in three scenarios, as made possible by the THOR 2 method. The results are presented in Table 3.

The high weight attributed to the Term criterion greatly impacted the result because the alternative Commercial Purchase (CC) is the one with the shortest term. On the other hand, the high weight attributed to the criterion ‘Adherence to business processes’ influenced the result of Scenario 2 because the first three are the ones with the best classification in this criterion. 

There is a contrast in the classifications of the alternatives for Commercial Purchase and Development by BN, which occupy first and fifth place in each scenario. This reflects that the assessment of the current situation experienced by BN is different from the market situation captured in the research conducted.

Both criteria have their advantages because, for BN, the value and customer satisfaction are in making a delivery in the shortest time, while the market, with an experience of successes and failures of implementation of HIS, already chooses to prioritize a deployment with more detailed requirements to ensure adherence to business processes.

### 3.2. PROMETHEE-SAPEVO-M1 Numerical Implementation

In implementing the THOR 2 method, we used a scale of scores to evaluate the alternatives for subjective approach criteria (Adherence, Customization, State of the Art and Vulnerability) and direct attribution for the criteria weights. Through the PROMETHEE-SAPEVO-M1 method, it is possible to operate a peer evaluation model between the alternatives in qualitative criteria and between the criteria, enabling the determination of a cardinal degree representing the importance of a given variable.

For the analysis of the alternatives in the criteria Adherence, Customization, State of the Art and Vulnerability, clarified as criteria with a qualitative approach, qualitative evaluation is used, as presented. Performing the analysis, it is possible to present the cardinal quantities in each criterion. A linear function with thresholds *q* and *p* is used for the remaining criteria evaluated quantitatively, maintaining the values assigned in the previous method. 

Using a similar approach to the determination of weights, a pairwise evaluation is performed between the criteria, first indicating a score and later a final weight for the aggregation of the model. The given evaluation is detailed in Table 4.

Once the criteria weights were obtained, a certain similarity and consistency were observed regarding the direct attributions performed in implementing the previous method; however, this clarifies the preferences similarly. With the established weights, the preferences are aggregated, enabling the global preference indexes, converted into positive flow, indicating the relative dominance of each alternative, negative flow, representing how much an alternative was dominated, and finally, the net flow, representing the final score and performing the ranking of the alternatives. The results generated are shown in Table 5.

With the net flows generated, a relative preference for the SL + FEM alternative was observed, presenting the highest aggregate score, thus establishing the CSC alternative in the second position of the ranking. Another relevant point is observed concerning the alternatives DBN and SL + BN, both presenting a relatively similar score, but considering only a small margin of error, which can establish a relationship of equivalence with each other.

Unlike the analysis performed previously by the THOR 2 method, it is observed that the DFEM and DBN alternatives were established in the last positions. However, it should be noted that the preference obtained by the DFEM alternative is similar to the S1 scenario of the previous evaluation. 

### 3.3. Comparison Analysis

The implementation of two distinct methodologies, based on the multi-criteria approach, aimed to establish a comparison relationship between the two analyses. The realization of a study where different methods are applied to a given set of data can be understood as a form of sensitivity analysis of the model, providing the manipulation of data and subjective elements by different approaches and mathematical techniques [58]. Similar studies are presented in [63,64,65].

The analysis performed using the THOR 2 and PROMETHEE-SAPEVO-M1 methods provided clarification of alternatives with good preference indexes in both implementations; in this context, the CSC and SL + FEM alternatives can be established as the most favorable forms of solution in the implementation of a HIS for BN, as shown in Table 6.

Another point to be discussed is linked to the feasibility of treating the subjectivity of the decision-taker in each evaluation. Considering the presence of quantitative and qualitative variables in the studied problem, both models enabled the implementation of different evaluation forms. In implementing the THOR method, we used a direct assignment for the criteria weights and a seven-point scale to evaluate the alternatives in qualitative criteria. On the other hand, in the PROMETHEE-SAPEVO-M1 method, the analysis of variables in a qualitative approach was performed by peer-to-peer evaluation, as established in the model [32]. The point of interest in a given analysis is treating personal information, clarifying the consistency of attributions in both models and detailing the preference relationships between alternatives in firstly a global and later a local character.

Both methods were favorable for a given implementation, providing an evaluation composed of data of different natures and treatment of subjective inputs. With the aggregation of preferences and obtaining the rankings of the solutions, it was possible to identify the most favorable changes in solving the problem, similarly clarifying forms of solution not favorable to the problem in question.

## 4. Discussion

The addressed study provided a comprehensive analysis concerning the evaluation of an alternative set under multiple criteria, exposing two favorable actions, the first, through the results of THOR 2 implementation, based on the purchase of commercial software (CSC) and the second, through the PROMETHEE-SAPEVO-M1 evaluation, related to internal development with free software with external foundation support (SL + FEM). Contextualizing decision-making in a high-level environment and envisioning a more favorable technological access to BN processes, the methodological approach allowed the treatment of data and evaluation of personal and deterministic information, respectively, leading to the preferences of the decision-makers for the sets of each variable established [66].

One of the main gains related to the methodological approach reflects the analysis performed under two types of distinct axiomatic models, providing different kinds of data manipulation, thus enabling the comparison between the results obtained and gaining robustness in the decision-making process.

It is emphasized that the given application of BN brought gains in mitigation in decision making, clarifying criteria that were not previously evaluated and exposing the forms of solution more adherent to the preferences established. Even working with two different methodologies, we highlight that they provided a similar result, where the alternatives CSC and SL + FEM were established as the better alternatives in each method analysis.

In search of an alternative point of view to the established evaluation, exploratory research was carried out in a market environment external to BN, making it possible to understand a scenario in a private technology organization. There was a change in preferences in the macro mode, thus presenting an aspect of divergence between public and private organizations. It should be emphasized that external research was not intended to change the established evaluation but to provide a new vision onto the subject and enable a future alignment of BN to the external technology market.

As a complementary method, we should emphasize that all the perspectives presented in the study are directly linked to the vision of a public military organization of the BN, and not representing the vision of the other Brazilian military forces. Another point in this decision scenario is presented by the hierarchy model of the military environment under analysis, where the final decision is not always in the powers of the direct managers of hospitals or technological development centers, these being often only advisors for a given high-level decision.

In this way, we can understand that BN should encourage an alignment between IT and its health sector to understand the opportunities for improvement of the current system and assess end-users’ needs in order to implement the appropriate improvements in the new solution. In parallel, this should be aligned with the organizational strategy, which is essential for a coherent evaluation of variables.

Some limitations are noted in the research in question as a contribution to the discussion. As a first point, it should be noted that qualitative assessments, based on subjective factors of decision-makers, do not yet have an analysis format to assess the consistency of assignments and this factor is axiomatic related to improvement of future studies. Finally, we emphasize that the study in question brings a perception of decision-making restricted to the evaluation of technologies for a healthcare management scenario for a military organization, thus being able to present differences in perceptions if the models are applied in other scenarios that use similar criteria.

## 5. Conclusions

Based on the scenario presented during this work concerning the feasibility of a HIS for the Brazilian Navy, the evaluation model obtained a comprehensive acceptance by the group of decision-makers. In the analysis, the system level of detail at the time of selection enabled the use of the information available. In addition, the methodology presented the possibility of immediate application of the developed model without the need to adapt internal processes, considering the requirements and restrictions of the problematic situation.

The application of two distinct methodologies based on the multi-criteria approach allowed parallel analyses in favor of clarifying favorable alternatives for a solution. It is noteworthy how important this is in arriving at a favorable determination, and it was also of great value in understanding non-favorable forms of the solution. Both models provided given implementations through their computational tools, providing a processing, treatment and trivial analysis of data and information of a variable character related to the models, enabling the exploration of the results in numerical and graphic fashion.

In addition, the analytical approach addressed the established objectives, allowing the organization to enable a HIS to BN in a structured and transparent way. It is noteworthy that the results obtained were relevant and proved, through the multiple forms of evaluations applied, the robustness of the models employed, enabling the expansion of the application of the outranking models to other areas of high-level decision-making regarding BN, necessary for the adjustment of variables to problematic situations.

In this context, it is considered that this choice is complex, involving multiple actors, consolidated organizational culture and their strategy. The study aims to contribute by bringing new perspectives on analysis, understanding obstacles faced by IT and hospitals and providing information for decision-making when choosing the implementation of a HIS. Thus, the application of THOR 2 and PROMETHEE-SAPEVO-M1 methods was robust and capable of ordering alternatives in all scenarios according to their criteria and decision-making factors, presenting coherent and concrete results for decision-making.

Considering the above study, the methodology presented in this research is highly replicable to support decision-making in the most diverse operational, tactical and strategic multidisciplinary problems. Therefore, this paper contributes to managerial, academic and social contexts in the public and private spheres.

As a form of future study, it is intended to increase the number of variables linked to the problems in question, providing new alternatives and criteria for analysis, linked to the possibilities of improvements to BN processes and providing the implementation of the multi-criteria approach in other management areas for given military organizations, enabling better decision-making analysis for macro-organizational development.

## Figures and Tables

**Table 1:** Decision Matrix.

	Cost	Time	Technological Dependence	Need for SL	Adherence	Customization	State of the Art	Vulnerability
CSC	6,280,131.22	12	100	4	2	2	7	3
SL + BN	583,989.83	78	40	20	4	4	5	6
SL + FEM	4,206,620.55	36	50	20	4	5	5	5
DBN	523,989.83	130	10	20	7	7	4	7
DFEM	12,702,048.10	60	20	20	7	7	4	6
DFST	16,837,941.22	60	30	8	6	6	5	5

**Table 2:** Criteria weights and thresholds.

Criteria	Weight	*p*	*q*
Cost	0.15	2,000,000.00	400,000.00
Time	0.25	24	6
Technological Dependence	0.05	30	10
Need for MO	0.10	8	4
Adherence	0.05	2	1
Customization	0.15	2	1
State of the art	0.15	2	1
Vulnerability	0.10	2	1

**Table 3:** Results of three scenarios by THOR 2 method.

Scenario 1		Scenario 2		Scenario 3	
CSC	2.850	CSC	3.23	CSC	3.28
SL + FEM	2.50	DFEM	2.08	DFEM	2.95
SL + BN	2.50	SL + FEM	2.00	SL + FEM	2.79
DFST	2.50	DFST	1.68	DFST	2.34
DFEM	2.00	DBN	1.50	DBN	1.91
DBN	2.00	SL + BN	1.00	SL + BN	1.31
CSC	2.850	CSC	3.23	CSC	3.28
SL + FEM	2.50	DFEM	2.08	DFEM	2.95

**Table 4:** Calculation of weights in the criteria in PROMETHEE-SAPEVO-M1.

	Adherence	Customization	State of the Art	Vulnerability	Cost	Time	Tech. Dependency	Need for MO		Maximum Sum = 21 Minimum Sum = −21				
										Punctuation		Normalized Punctuation		Final Weights
Adherence	0	−2	−2	−1	−2	−3	0	−1	=	−11	=	0.238095	=	0.060
Customization	2	0	0	1	0	−1	1	1	=	4	=	0.595238	=	0.149
State of the art	2	0	0	1	0	−1	1	1	=	4	=	0.595238	=	0.149
Vulnerability	1	−1	−1	0	−1	−3	1	0	=	−4	=	0.404762	=	0.101
Cost	2	0	0	1	0	−1	2	1	=	5	=	0.619048	=	0.155
Time	3	1	1	3	1	0	3	3	=	15	=	0.857143	=	0.214
Tech. dependency	0	−1	−1	−1	b	−3	0	1	=	−5	=	0.380952	=	0.083
Need for MO	1	−1	−1	0	−1	−3	−1	0	=	−6	=	0.357143	=	0.089

**Table 5:** Importance of flows and ranking obtained in the PROMETHEE-SAPEVO-M1 method.

Alternatives	Positive Flow	Negative Flow	Net Flow
SL + FEM	0.217	0.169	0.048
CSC	0.283	0.260	0.023
DBN	0.197	0.203	−0.006
SL + BN	0.167	0.174	−0.007
DFST	0.172	0.185	−0.013
DFEM	0.169	0.204	−0.045

**Table 6:** Final ranking in both methods.

THOR Final Ranking	PROMETHEE-SAPEVO-M1 Final Ranking
CSC	SL + FEM
DFEM	CSC
SL + FEM	DBN
DFST	SL + BN
DBN	DFST
SL + BN	DFEM

## Data Availability

Not applicable.
